# Immunization with a MOMP-Based Vaccine Protects Mice against a Pulmonary *Chlamydia* Challenge and Identifies a Disconnection between Infection and Pathology

**DOI:** 10.1371/journal.pone.0061962

**Published:** 2013-04-16

**Authors:** Connor P. O’Meara, Charles W. Armitage, Marina C. G. Harvie, Peter Timms, Nils Y. Lycke, Kenneth W. Beagley

**Affiliations:** 1 Institute of Health and Biomedical Innovation (IHBI), Queensland University of Technology (QUT), Brisbane, Queensland, Australia; 2 Mucosal Immunobiology and Vaccine Centre (MIVAC), University of Göteborg, Göteborg, Götaland, Sweden; Instituto Butantan, Brazil

## Abstract

*Chlamydia pneumoniae* is responsible for up to 20% of community acquired pneumonia and can exacerbate chronic inflammatory diseases. As the majority of infections are either mild or asymptomatic, a vaccine is recognized to have the greatest potential to reduce infection and disease prevalence. Using the *C. muridarum* mouse model of infection, we immunized animals via the intranasal (IN), sublingual (SL) or transcutaneous (TC) routes, with recombinant chlamydial major outer membrane protein (MOMP) combined with adjuvants CTA1-DD or a combination of cholera toxin/CpG-oligodeoxynucleotide (CT/CpG). Vaccinated animals were challenged IN with *C. muridarum* and protection against infection and pathology was assessed. SL and TC immunization with MOMP and CT/CpG was the most protective, significantly reducing chlamydial burden in the lungs and preventing weight loss, which was similar to the protection induced by a previous live infection. Unlike a previous infection however, these vaccinations also provided almost complete protection against fibrotic scarring in the lungs. Protection against infection was associated with antigen-specific production of IFNγ, TNFα and IL-17 by splenocytes, however, protection against both infection and pathology required the induction of a similar pro-inflammatory response in the respiratory tract draining lymph nodes. Interestingly, we also identified two contrasting vaccinations capable of preventing infection or pathology individually. Animals IN immunized with MOMP and either adjuvant were protected from infection, but not the pathology. Conversely, animals TC immunized with MOMP and CTA1-DD were protected from pathology, even though the chlamydial burden in this group was equivalent to the unimmunized controls. This suggests that the development of pathology following an IN infection of vaccinated animals was independent of bacterial load and may have been driven instead by the adaptive immune response generated following immunization. This identifies a disconnection between the control of infection and the development of pathology, which may influence the design of future vaccines.

## Introduction

Serological evidence suggests that 80% of people will contract a *C. pneumoniae* respiratory tract infection at one point in their lifetime [1]. In addition to respiratory tract infections, *C. pneumoniae* infections are implicated in the exacerbation of cardiovascular disease, asthma, chronic obstructive pulmonary disease, multiple sclerosis, Alzheimer’s disease and reactive arthritis [2], [3]. Many of these diseases have multi-billion dollar healthcare expenditures and are leading causes of morbidity and mortality in most nations. Confounding the matter of infection control, most acute *C. pneumoniae* respiratory infections are difficult to diagnose and treat [4], [5]. Furthermore, around 75% of first infections occur between the ages of 5–14 years [6], highlighting the need for early intervention to prevent infection and the potential predisposition/exacerbation of chronic inflammatory diseases. Consequently, development of a vaccine is most logical solution suited to controlling the spread of infection.

*Chlamydiae* are obligate intracellular pathogens that infect through and predominantly reside in the mucosal epithelium. Protection against infection therefore, is reliant on the induction of a mucosal immune response at the anatomical portal of entry of the invading pathogen [7]. Vaccines targeted to mucosal epithelia, without the necessity for needles, elicit mucosal immunity by stimulating local innate cell populations that are preconditioned to generate adaptive immune responses at mucosal surfaces. However, as immunological tolerance is often the default response to mucosal antigen exposure, any mucosal vaccine must overcome this in order to elicit robust and long-lived mucosal immunity. Unfortunately, a major obstacle to the development of an effective vaccine for *Chlamydia*, and other mucosal pathogens, is the lack of potent adjuvants proven to elicit protective responses at mucosal surfaces.

There is a need for further research and development of new mucosal adjuvants, however, vaccine safety cannot be compromised. In 2001, the mucosal vaccine Nasalflu*®* was withdrawn from the market due to facial paresis (Bell’s palsy) developing in some recipients. This was later attributed to toxicity of the enterotoxic adjuvant (heat-labile toxin – LT, a bacterial toxin derived from *Escherichia coli*) when administered by the nasal route [8]. Interestingly, the same adjuvant applied topically can be equally immunogenic without the inherent toxicity [9]. This shows that utilizing different routes of immunization can limit the toxicity associated with certain adjuvants like LT and CT, whilst still harnessing their immunomodulatory benefits.

The detoxification of the enterotoxins such as CT and LT is also a growing area of research. This is because the nasal route of immunization is still of particular interest for human vaccines designed to protect against mucosal pathogens. CTA1-DD consists of the enzymatically active CTA1 subunit of CT, genetically linked to a dimer of an immununoglobulin (Ig)-binding domain (D) from the staphyloccocal protein A [10]. Inclusion of the DD moiety targets the adjuvant’s immunogenic activity primarily to B cells expressing surface Ig [11]. As a result, CTA1-DD retains the adjuvanticity of the native holotoxin, but most importantly, is up to 1000-fold less toxic in primates when given IN [12]. Promoting both antibody and cell-mediated responses, CTA1-DD has been shown to enhance protection against a number of different viral and bacterial mucosal pathogens [12]–[18]. CTA1-DD therefore represents a new generation of non-toxic mucosal adjuvants. The ability of CTA1-DD however to confer protection against a chlamydial respiratory tract infection and the associated pathology is yet to be investigated.

CT/CpG, when combined with MOMP, has been shown to elicit good protection against a respiratory tract chlamydial challenge [19] and can be used for comparison with the new generation adjuvant CTA1-DD. Despite the potential for toxic side effects of the CT/CpG combination if administered IN, the same adjuvant combination applied via SL or the TC routes can be equally immunogenic in animal models [19], [20], without the inherent toxicity. In the present study we compared two adjuvants, CTA1-DD and the CT/CpG combination, together with MOMP. Animals were vaccinated with these two different vaccines via three different needle-free routes of immunization (IN, SL and TC), chosen for their abilities to elicit mucosal immunity in the lungs [21]. Following immunization, the induction of systemic and mucosal immune responses were quantified and compared with the protection conferred against infection and pathology following a respiratory tract challenge with *C. muridarum*.

## Methods

### Ethics statement

This study was approved by the Queensland University of Technology Animal Ethics Committee (Approval number 0800000432) and carried out in strict accordance with any recommendation. All procedures were performed under anesthesia, and all efforts were made to minimize suffering.

### MOMP recombinant and Chlamydia muridarum purification

Recombinant *C. muridarum* MOMP was purified from the *E. coli* (DH5α[pMMM3]) clone transformed with the pMAL-c2 ampicillin-resistant vector encoding the recombinant maltose-binding protein fusion protein (MOMP-MBP) as previously described [19]. Endotoxin levels were quantified using ToxinSensor*®* chromogenic *Limulus* amebocyte lystate (Genscript – Life Research, VIC, Australia) and reduced using DetoxiGel*®* (Thermo Fischer Scientific, IL, USA) to 0.044EU/mL, below that capable of stimulating an immune response by the IN route [11]. *C. muridarum* (Weiss strain; ATCC VR-123, VA, USA), formerly mouse pneumonitis biovar of *C. trachomatis*, was cultured and purified as previously described [22].

### Immunization protocols

Mice were sourced from the Animal Resource Centre (WA, Australia) at 6 weeks of age. Mice were immunized on days 0, 7, 14 and 28 via either IN, TC or SL routes. This immunization schedule was chosen because it will allow a retrospective comparison between past and present work [18], [19], [23]–[25]. Animals in the IN group were lightly anesthetized with 4%v/v isofluorane (Abbott Australasia, NSW, Australia). The anesthetized mice were placed on their backs, held at a downward angle then immunized with MOMP (100 µg) and either CTA1-DD (MIVAC, The University of Göteborg) (20 µg) or CT (List Biological Laboratories – Sapphire Biosciences, NSW, Australia) (5 µg) plus CpG-ODN 1826*^c^* (5′-TCC ATG ACG TTC CTG ACG TT-3′) (Sigma-Aldrich, NSW, Australia) (10 µg) mixed in a 10 µL volume, 5 µL applied to each nare. Animals in the TC group were anesthetized with an intraperitoneal (IP) injection of ketamine (100 mg/kg) (Parnell Laboratory, NSW, Australia) and xylazine (10 mg/kg) (Bayer, NSW, Australia). A 1.5 cm^2^ area of skin on the back of mice at the base of the tail was shaved using clippers, with care taken not to break the skin. The skin was pre-treated first with acetone, then with a solution containing dodecylpyridinium chloride (DPC) (0.33%w/v), isopropyl myristate (IPM) (0.33%w/v) and methyl pyrolidone (MPR) (0.33%w/v) [26] and then rehydrated with phosphate buffered saline (PBS). Mice then received granulocyte-macrophage colony-stimulating factor (GM-CSF) (Invitrogen, VIC, Australia) (12.5 ng) [25] with MOMP (200 µg) and either CTA1-DD (20 µg) or CT (10 µg) plus CpG-ODN 1826*^c^* (10 µg) mixed in a volume of 50 µL. The immunization was contained for a 24 hr time period using a patch system consisting of gauze, Opsite Flexifix*®* (Smith & Nephew, QLD, Australia) and Micropore*®* surgical tape (3M, QLD, Australia). The SL group was also anesthetized with ketamine and xylazine. MOMP (100 µg) mixed with either CTA1-DD (20 µg) or CT (5 µg) plus CpG-ODN 1826*^c^* (10 µg) in a 7 µL volume was applied directly to the ventral side of the tongue and left for 1 hr with the head of the mice maintained in ante-flexion. The antigen and adjuvant dosages used for each route of immunization were selected by their ability to elicit optimal antigen-specific cell-mediated and humoral responses (data not shown). Each route included antigen (MOMP) alone, adjuvant alone (CTA1-DD or CT/CpG) and unimmunized (PBS) control groups. Animals were euthanized 7 days after their final boost using Lethabarb*®* (200 mg/kg) (Virbac, NSW, Australia) delivered IP.

### Lymphocyte proliferation and cytokine analysis

The spleen and mediastinal lymph nodes (MdLN) of mice were mechanically disrupted through 70 µm nylon filters (BD Bioscience, NSW, Australia). The red blood cells were lysed using red blood lysis buffer (155 mM NH_4_Cl, 12 mM NaHCO_3_, 100 µM EDTA, pH 7.35) for 5 min on ice. Lymphocytes from each tissue were resuspended in complete Dulbecco’s minimal essential medium (DMEM) (5%v/v fetal calf serum (FCS), 4 mM L-glutamine, 50 µg/mL gentamycin, 100 µg/mL streptomycin sulfate) (Invitrogen) containing 50 µM β-mercaptoethanol and seeded into a U-bottom 96-well plates (5×10^5^ cells/well). Lymphocytes were then supplemented with either normal media (unstimulated) or stimulated with media containing MOMP (10 µg/well) for 72 hr at 37°C with 5% CO_2_. Following the incubation, 100 µL of media was removed and stored at −80°C for analysis of antigen-specific cytokine production. Cells were then incubated with 0.5 µCi ^3^H-thymidine/well for an additional 14 hr time period. The incorporation of ^3^H-thymidine was assessed using a Wallac 1450–030 MicroBeta TriLux Liquid Scintillation and Luminescence Counter (PerkinElmer, VIC, Australia). Results were expressed as a stimulation index; dividing counts per minute (cpm) of the MOMP stimulated cultures by the cpm of the media background.

Pooled supernatants from stimulated immune cell preparations were analyzed for levels of IFNγ, IL-4, IL-10 and IL-17 using a customized BioPlex Mouse Express Pro Cytokine Assay 4-plex (Biorad Laboratories – Life Sciences, VIC, Australia) according to the manufacturer’s instructions and read on a Bioplex-200 system with Luminex xMap*®* technology (Biorad Laboratories). TNFα levels were also detected from the pooled supernatant using the DuoSet ELISA development system (R&D systems – Sapphire Biosciences) according the manufacturer’s instructions.

### MOMP-specific antibodies

MOMP-specific antibodies were quantified by ELISA as previously described [19]. Briefly, 96-well plates were incubated overnight with 2 µg/well of MOMP in 50 µL of borate buffered saline (0.5M boric acid, 1.5M NaCl, pH 8.4). The following day plates were washed with PBS 0.05%v/v Tween20*®* (PBST) and then blocked with 5%v/v FCS, PBST for 2 hr at 37°C. Samples were serially diluted in the MOMP-coated plate using PBST and incubated at 37°C for 1 hr. Plates were washed thoroughly with PBST and then incubated with rabbit horseradish peroxidase (HRP)-conjugated anti-mouse IgA, IgG, IgG1 and IgG2a (Southern Biotech – In vitro Technologies, VIC, Australia). The substrate, 3,3,5,5-tetramethylbenzidine in a phosphate citrate buffer (Sigma-Aldrich), was added to each well and incubated for approximately 10 min at room temperature. The reaction was stopped by the addition of 1M H_2_SO_4_ and the OD_450nm_ was measured on an xMark*®* microplate spectrophotometer (Biorad Laboratories). Endpoint titers were calculated for all samples using non-linear regression analysis and background absorbance of PBST plus two standard deviations (SD). Only those animals vaccinated with the antigen induced MOMP-specific antibodies, whereas those in unimmunized and adjuvant only control groups did not (data not shown).

### In vitro neutralization of C. muridarum infection

Lavage and serum samples, diluted 1/10 in complete DMEM, were incubated with 10^3^ inclusion-forming units (IFUs) of purified *C. muridarum* in a 100 µL volume for 1 hr at 37°C with 5% CO_2_. This suspension was then applied to confluent McCoy cell monolayers in a 48-well plate and incubated for 3 hr. Following incubation the antibody/*Chlamydia* suspension was removed, the monolayer washed with PBS and replaced with media containing 1 µg/mL of cycloheximide. The infection was stopped after 24 hr by fixing with methanol for 10 min. Each well was blocked overnight at 4°C with a solution containing 5%v/v FCS, 0.05%w/v sodium azide and PBST. Cells were then incubated with a 1/500 dilution of a sheep anti-*C. muridarum* recombinant MOMP-specific polyclonal antibody (produced by the Institute of Medical and Veterinary Science, SA, Australia, as previously described [19]) in blocking solution for 1 hr at 37°C. Each well was rinsed thoroughly with PBST following incubation, then stained with 1 µg/mL of donkey anti-sheep AlexaFluor-488 secondary antibody (Invitrogen) and 10 ng/mL 4',6-diamidino-2-phenylindole, dihydrochloride (DAPI) (Invitrogen) for 1 hr at 37°C. Each well was then washed with PBST and inclusions were enumerated by fluorescent microscopy. Images from three random fields of view were taken from each well using a Nikon Eclipse TE2000-U fluorescent inverted microscope fitted with a Nikon Digital Eclipse DXM 1200C camera (Nikon Australia, NSW, Australia). Chlamydial inclusion and host cell nuclei were counted simultaneously using MetaMorph Imaging Series 7.6 software (Molecular Devices – SDR Clinical Technology, NSW, Australia). Percentage neutralization was determined using the equation % neutralization  =  [% cells infected (post-immunization) - % cells infected (pre-immunization)] / % cells infected (no sample – media).

### Intranasal C. muridarum challenge and monitoring

The respiratory tract challenge was conducted as previously described [19]. Briefly, seven days after the final boost animals were challenged IN with 10^3^ IFU of purified *C. muridarum*, in a 10 µL volume, 5 µL applied to each nare. The live infection control group was infected six weeks prior to re-challenge. All animals were weighed daily to monitor the development of a lung infection indirectly through cachexia [27].

### Quantification of chlamydial burden in the respiratory tract tissues

Quantification of the recoverable amount of *Chlamydia* from lung homogenates was performed as previously described [19]. The lungs from each mouse were homogenized in 320 µL of sucrose phosphate glutamine (219 mM sucrose, 3.8 mM KH_2_PO_4_, 8.6 mM Na_2_HPO_4_ and 4.9 mM glutamic acid, pH 7.0) using an OMNI TH tissue homogenizer with the 7 mm saw-toothed stainless steel attachment (OMNI International, GA, USA). Total genomic DNA was extracted from an overnight proteinase K digest of 50 µL of tissue homogenate using the Wizard Genomic DNA Purification Kit (Promega, NSW, Australia) according to the manufacturer’s instructions. Quantitative real-time PCR (qRT-PCR) was used to determine *C. muridarum* DNA levels in total mouse lung genomic DNA using a standard curve generated from known copies of the outer membrane protein A (*omp*A) PCR product. The primer sequence used in the qRT-PCR amplification of *omp*A gene encoding the MOMP of *C. muridarum* is as follows: 5′-GCC GTT TTG GGT TCT GCT T-3′ and 5′-CGA GAC GTA GGC TGA TGG C-3′ (Sigma-Aldrich). Each reaction contained a final concentration of 1 µM of forward/reverse primers, 200 µM dNTP’s, 1.5 mM MgCl_2_, 1X buffer, 0.15X SYBR green, and 5U of Platinum Taq polymerase (Invitrogen) made up to a final 20 µL volume using sterile endonuclease-free water. Cycling conditions were 95°C denaturation for 20 sec, followed by a 64°C annealing for 20 sec and a 72°C extension for 15 sec repeated for a total of 30 cycles. qRT-PCR was performed using the Corbet Rotorgene Q (QIAGEN, VIC, Australia).

### Histopathology

The lungs from each mouse were excised and preserved by fixing in 70%v/v ethanol. Fixed tissues were then imbedded in paraffin, sectioned (5 µm) and stained with Masson’s trichrome for evaluation of fibrosis (QML Diagnostics, QLD, Australia). Sections were scored, by three individuals in a blinded fashion, based on inflammation and scaring of the lung tissue (Table 1). The final results were the average of all scores.

**Table 1:** Histopathological scoring system for mouse lungs.

Fibrosis score	Description	Inflammation
0	Lack of fibrosis around tissue	Absent
1	Some fibrosis around tissue	Mild
2	Some tissue has significant fibrosis or majority of tissue has some fibrosis	Moderate
3	The majority of tissue has significant fibrosis	Severe

### Statistical analysis

All data is presented as the mean ± SD. All statistics were performed using GraphPad Prism*®* version 5.00 (GraphPad Software Inc, CA, USA). Significant differences were determined using a one-way analysis of variance (ANOVA) with Tukey’s post-test. Significance was set at *P*<0.05 for all tests. *P*>0.05 (not shown), 0.01–0.05 (*), 0.001–0.01 (**) and <0.001 (***).

## Results

### Protection against infection following live C. muridarum intranasal challenge

**Figure 1:**
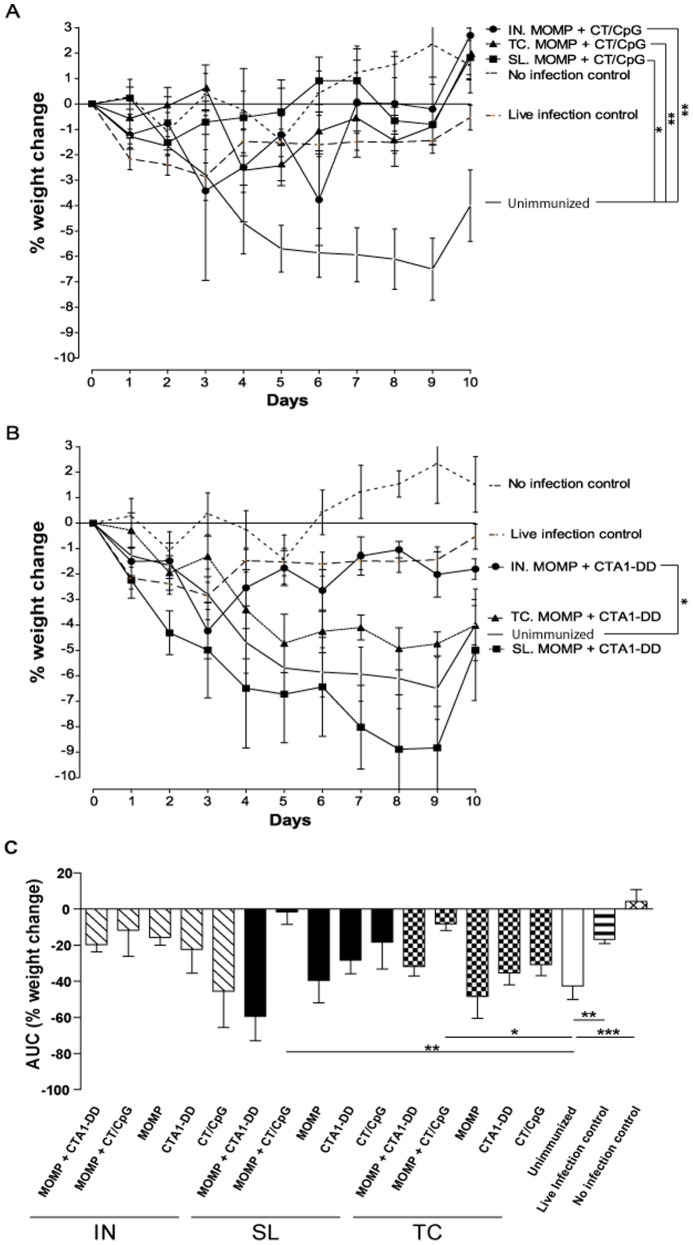
Percentage weight change of animals following IN challenge with *C. muridarum*. Percentage weight change was calculated by comparing the pre-infection body weight to daily p.i body weights. The figure depicts the effect of vaccination with MOMP and (A) CT/CpG- or (B) CTA1-DD-based vaccines on weight loss following IN challenge infection. Unimmunized (primary infection), live infection (secondary infection) and no infection controls are also included. (C) Area under the curve (AUC) analysis of percentage weight change was the total area, of both negative and positive peaks in weight change, over the 10 day course of infection in arbitrary units. Results are presented as the mean ± SD. Significant differences were determined using a one-way ANOVA with Tukey’s post-test by comparing the weight changes between groups at the same point in time. One *P* value, the most significant, is given for groups showing a significant change. Significance was set at *P*<0.05 for all tests. *P*>0.05 (not shown), 0.01–0.05 (*), 0.001–0.01 (**) and <0.001 (***).

Firstly, we assessed whether either vaccine was capable of preventing the development of pneumonia indirectly through cachexia. Unimmunized animals developed severe cachexia following an IN infection (Figure 1). The percentage weight loss in unimmunized animals peaked on day 9 p.i at approximately 7%. The live infection control animals that had recovered from a primary infection were strongly protected against weight loss following re-infection. This group began regaining weight by day 4 p.i, significantly faster than the unimmunized animals at day 10 p.i (*P*<0.001). The no infection control group had a net positive weight gain over the 10 day time period, which was significant when compared to infected control animals (*P*<0.001) and indicated that cachexia was absent in uninfected animals. Immunization with MOMP and CT/CpG, by any route, significantly protected animals from the weight loss associated with a chlamydial respiratory tract challenge (*P*<0.05–0.01) (Figure 1A). SL and TC immunization with MOMP and CT/CpG also provided a significant level of protection from the overall magnitude of cachexia over the duration of the infection when compared to the antigen, adjuvant and unimmunized controls (*P*<0.05–0.01) (Figure 1C). Interestingly, only IN immunization with MOMP and CTA1-DD significantly prevented weight loss (*P*<0.05) when compared to the unimmunized control (Figure 1B). The protection against weight loss conferred by this vaccination was equivalent to the live infection control group. IN immunization with MOMP and CTA1-DD also induced a comparable level of protection against weight loss as MOMP and CT/CpG delivered by the same route. Neither SL nor TC immunization with MOMP and CTA1-DD prevented the development of cachexia following infection.

**Figure 2:**
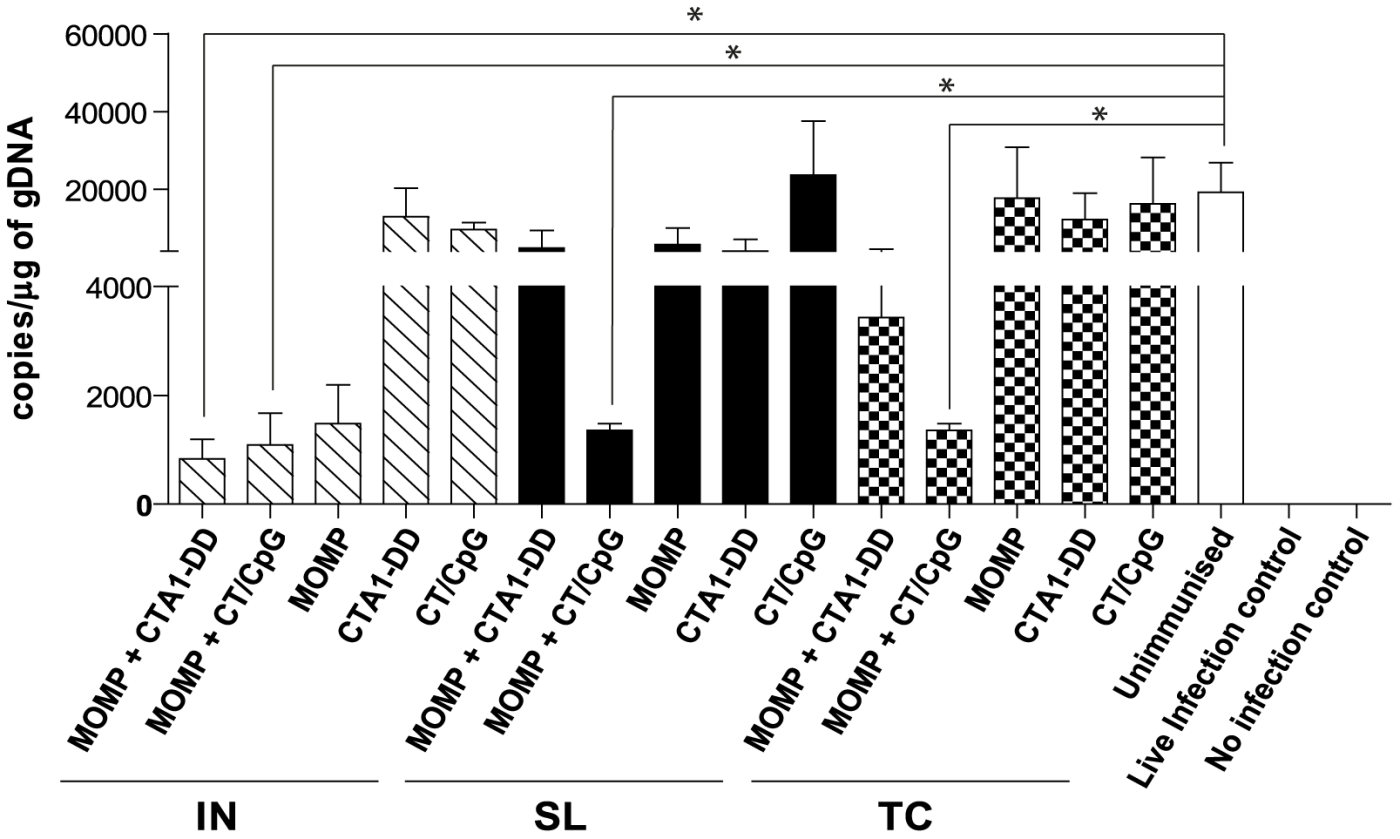
Chlamydial burden in lung tissue at day 10 p.i determined by *omp*A PCR. Genomic DNA (gDNA) was extracted from lung tissues collected at day 10 p.i. Chlamydial DNA was quantified from lung gDNA by *omp*A-specific qRT-PCR using a standard curve. The copies of *C. muridarum* DNA per µg of host gDNA for each vaccine were grouped with their respective route of immunization. Results are presented as the mean ± SD. Significant differences were determined using a one-way ANOVA with Tukey’s post-test. Significance was set at *P*<0.05 for all tests. *P*>0.05 (not shown), 0.01–0.05 (*), 0.001–0.01 (**) and <0.001 (***).

The bacterial burden in the lung tissues was then quantified from each group to validate cachexia results. All unimmunized animals were PCR positive for *C. muridarum* following challenge (Figure 2), with bacterial loads in excess of 4×10^4^ copies/µg of gDNA. The live infection control group was negative for chlamydial DNA at day 10 p.i. This absence of infection in the live infection control group supports the strong resistance to re-infection indicated by the weight loss data (Figure 1). No chlamydial DNA could be detected in the uninfected control group. The vaccinations that were protective against weight loss also greatly reduced the bacterial burden in the lungs at the peak of infection (Figure 2). Immunization with MOMP and CT/CpG, by IN, SL and TC routes, significantly reduced levels of recoverable bacterial DNA when compared to the unimmunized controls (*P*<0.05). Conversely, MOMP and CTA1-DD was only protective following IN administration (*P*<0.05). TC and SL immunization with MOMP and CTA1-DD, which failed to prevent weight loss (Figure 1), also induced no significant reduction in bacterial DNA (Figure 2).

### Protection against pathology following live C. muridarum intranasal challenge

**Figure 3:**
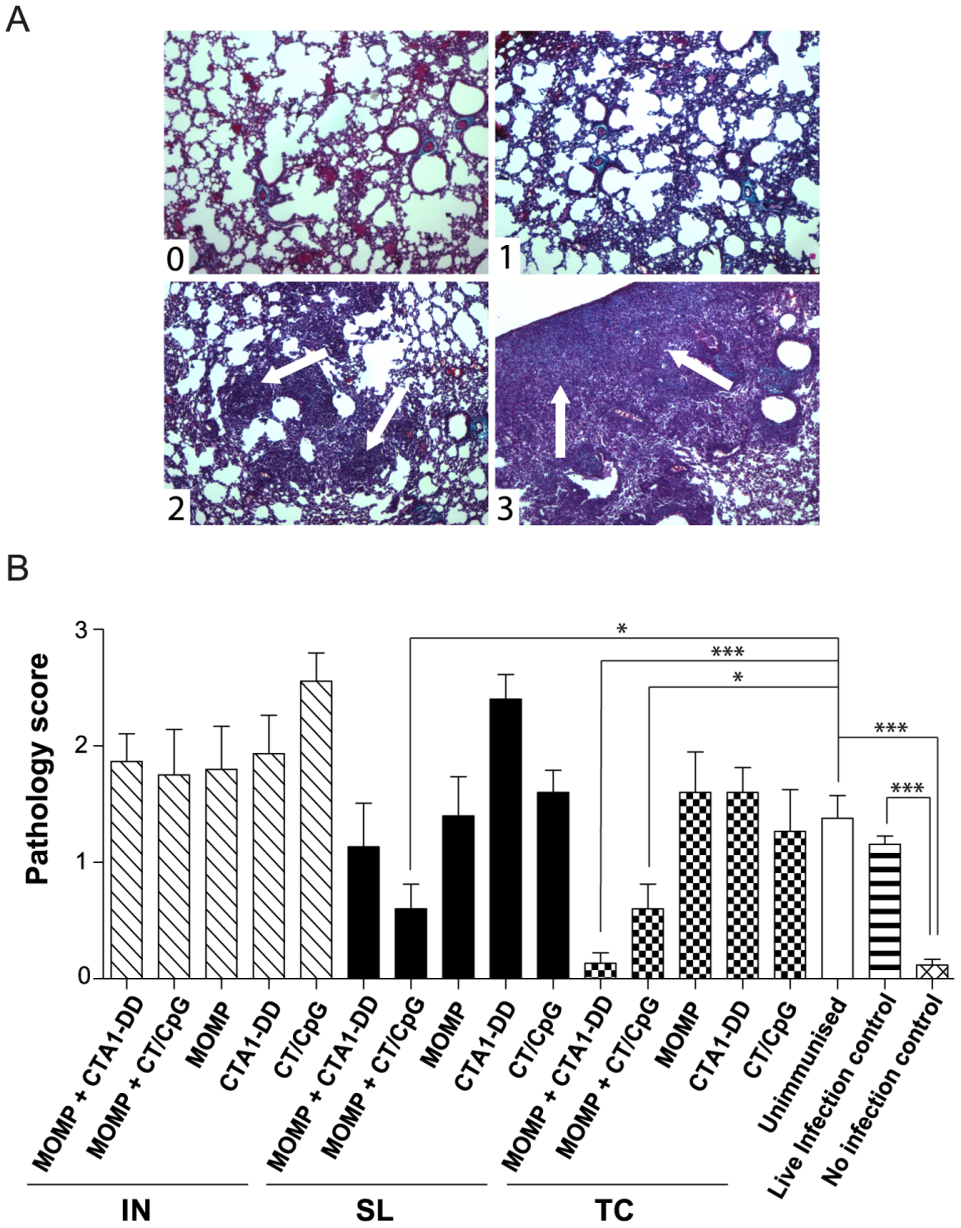
Fibrotic scarring and lung consolidation following IN infection with *C. muridarum*. Lung tissues collected at day 10 p.i were paraffin embedded, sectioned and stained using Masson’s trichrome. (A) The pathology score scale used to compare the development of disease following infection. Representative histological images for each pathology scores are depicted. “0” signifies healthy, undamaged lung tissue. Scores of “1” through to “3” depict worsening degrees of collagen deposition (blue) and the beginnings of obstruction and consolidation of the lungs (white arrows). Pathology can also be seen on a macroscopic level, when comparing healthy (left) and fibrotic (right) lobes of the lungs. (B) Average pathology scores, determined by three separate individuals blinded to groups and experimental design. Results are presented as the mean ± SD. Significant differences were determined using a one-way ANOVA with Tukey’s post-test. Significance was set at P < 0.05 for all tests. P >0.05 (not shown), 0.01–0.05 (*), 0.001–0.01 (**) and <0.001 (***).

We next determined whether either vaccine was capable of preventing the fibrotic scarring associated with an active chlamydial pulmonary infection. Lung tissues taken from infected, unimmunized animals showed significant collagen deposition and lung consolidation (Figure 3A). The live infection control group, strongly resistant to re-infection (Figure 1 and 2), showed a similar level of fibrosis in the lungs to the naive unimmunized animals (Figure 3B). The mock infection control group displayed no evidence of fibrotic scarring when compared to the infection controls (unimmunized and live infection control) (*P*<0.001). Animals immunized with MOMP and CT/CpG via the TC and SL routes, which were shown to provide protection against cachexia and the lung chlamydial burden (Figure 1 and 2), were also significantly protected from pathology when compared to the unimmunized controls (*P*<0.05) (Figure 3B). Neither vaccine delivered IN protected against pathology (Figure 3B), despite reducing weight loss and bacterial burden (Figure 1 and 2). The greatest protection against fibrotic scarring however was seen in animals immunized by the TC route with MOMP and CTA1-DD, where the pathology score of this group was equivalent to that of uninfected animals (Figure 3B). Interestingly, these animals were not significantly protected against weight loss or chlamydial burden (Figure 1 and 2).

### Antigen-specific proliferation and cytokine production by splenocytes

**Figure 4:**
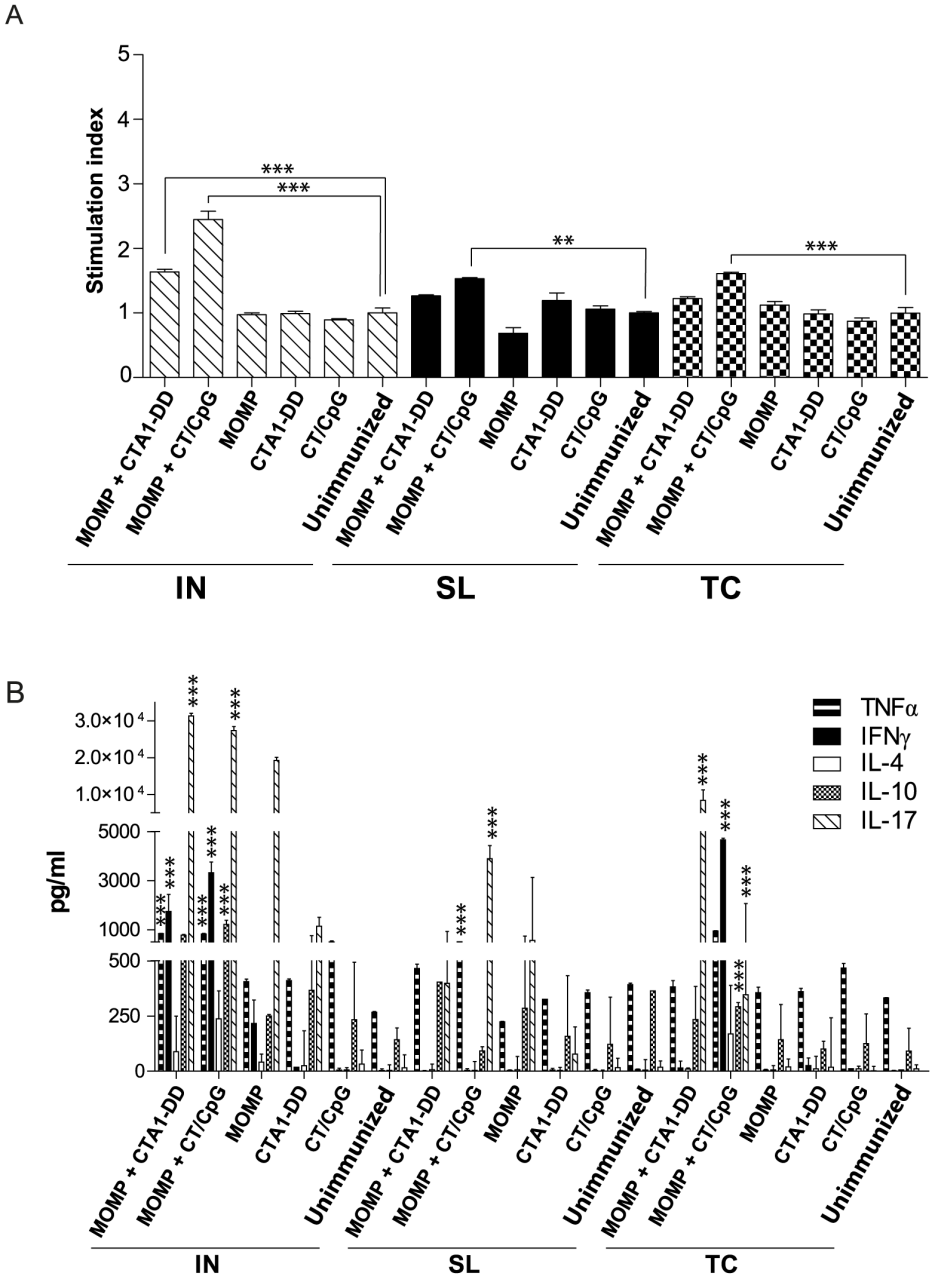
Proliferation and cytokine production by lymphocytes isolated from the spleen and stimulated with MOMP. Splenocytes were stimulated with MOMP or media for 72 hr. (A) Lymphocyte proliferation was determined by addition of thymidine for an additional 14 hr. Data are expressed as stimulation index (cpm of the MOMP stimulated cells divided by the cpm of media stimulated cells). (B) The amount of TNFα, IFNγ, IL-4, IL-10 and IL-17 (pg/mL) secreted by splenocytes following stimulation was quantified using Bioplex*®* and ELISA. Results are presented as the mean ± SD. Significant differences were determined using a one-way ANOVA with Tukey’s post-test. Significance was set at *P*<0.05 for all tests. *P*>0.05 (not shown), 0.01–0.05 (*), 0.001–0.01 (**) and <0.001 (***).

As protection against a chlamydial infection is predominantly driven by the cell-mediated response [28], we examined antigen-specific proliferation and cytokine secretions by lymphocytes isolated from the spleen and MdLN following *in vitro* re-stimulation with MOMP. IN immunization with either vaccine (MOMP and CTA1-DD or CT/CpG) elicited a significant increase in MOMP-specific proliferation by splenocytes when compared to the unimmunized control group (*P*<0.001) (Figure 4A). MOMP-specific proliferation was also significantly elevated in animals immunized with MOMP and CT/CpG via either the SL (*P*<0.01) or TC routes (*P*<0.001) when compared to the unimmunized controls.

Cytokines were only detected above background levels when splenocytes were shown to proliferate significantly in response to MOMP stimulation (Figure 4B). Immunization MOMP and CT/CpG, by any route, resulted in significant increase in cytokine secretions following *in vitro* stimulation with MOMP. The MOMP and CTA1-DD vaccine induced antigen-specific cytokine secretions by splenocytes when delivered via the IN and TC routes. Production of IFNγ was equal in animals receiving either IN delivered vaccine, but this was significantly more in mice TC immunized with MOMP and CT/CpG (*P*<0.05). IFNγ was only secreted by stimulated splenocytes isolated from animals IN immunized with MOMP and CTA1-DD. A significant increase in secretions of TNFα were also detected in all groups immunized with MOMP and CT/CpG regardless of route (*P*<0.001). Immunization with MOMP and CTA1-DD primed splenocytes to secrete a significant amount of TNFα following re-stimulation, but only when the vaccine was delivered IN (*P*<0.001). Antigen-specific IL-17 production was highest in IN vaccinated animals and was equivalent between adjuvants. With the exception of the SL immunization with MOMP and CTA1-DD, IL-17 was also secreted by splenocytes isolated from all other vaccine groups, but at lower levels than in IN immunized groups. Immunization with MOMP and CTA1-DD via the TC route only induced a significant amount of IL-17 (*P*<0.001). With the exception of the IN immunization with MOMP and CT/CpG (*P*<0.001), no vaccine induced a significant amount of IL-10. IL-4 was not detected in any vaccine group at levels above unimmunized controls.

### Antigen-specific proliferation and cytokine production by lymphocytes isolated from the MdLN

**Figure 5:**
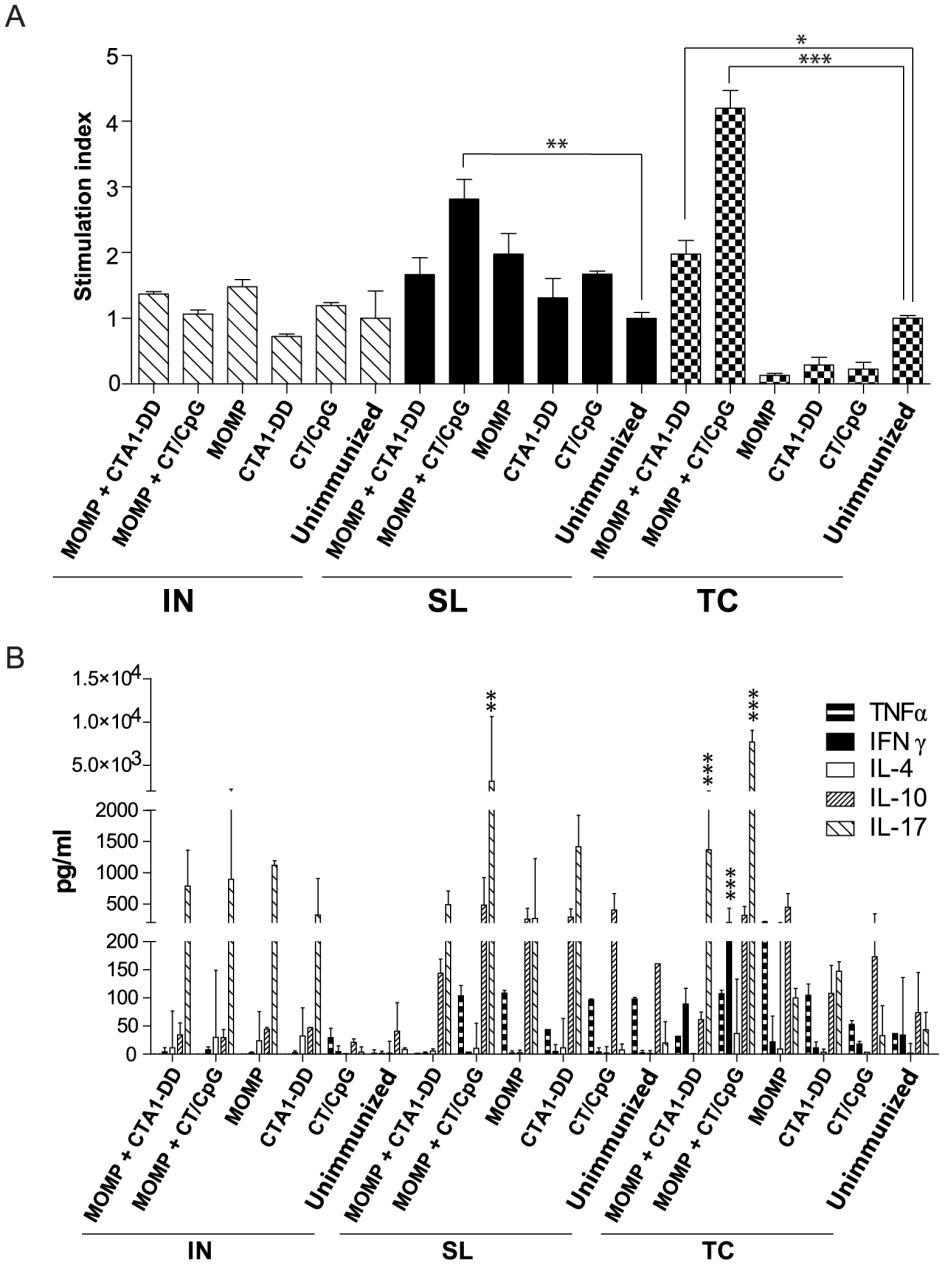
Proliferation and cytokine production by lymphocytes isolated from the MdLN and stimulated with MOMP. Lymphocytes isolated from the MdLN draining the lungs were stimulated with MOMP or media for 72 hr. (A) Lymphocyte proliferation was determined by incubating the stimulated cells with thymidine for an additional 14 hr and expressed as stimulation index. (B) The amount of TNFα, IFNγ, IL-4, IL-10 and IL-17 (pg/mL) secreted by the lymphocytes were quantified using Bioplex*®* and ELISA. Results are presented as the mean ± SD. Significant differences were determined using a one-way ANOVA with Tukey’s post-test. Significance was set at *P*<0.05 for all tests. *P*>0.05 (not shown), 0.01–0.05 (*), 0.001–0.01 (**) and <0.001 (***).

Immunization with MOMP and CT/CpG via the SL (*P*<0.01) or TC (*P*<0.001) routes, but not via the IN route, generated an antigen-specific lymphoproliferative response in the MdLN, significantly greater than unimmunized animals (Figure 5A). TC immunization with MOMP and CTA1-DD also induced a proliferative response in the MdLN, significantly higher than control animals (*P*<0.05), but not when the vaccine was delivered via the IN or SL routes.

Elevated levels of cytokine secretions were only detected in those groups previously shown to have significantly increased proliferation in the MdLN in response to MOMP stimulation (Figure 5B). This included the animals immunized with either vaccine by the TC route with MOMP and both adjuvant and the group SL immunized with MOMP and CT/CpG, groups that were also protected against pathology (Figure 3B). Lymphocytes isolated from these groups predominantly secreted IL-17 following stimulation. IFNγ was only detected in groups immunized via the TC route, although significance was limited to the animals immunized with MOMP and CT/CpG. TNFα, IL-4 and IL-10 were not found to be significantly elevated in any group.

### Antigen-specific serum antibodies

**Figure 6:**
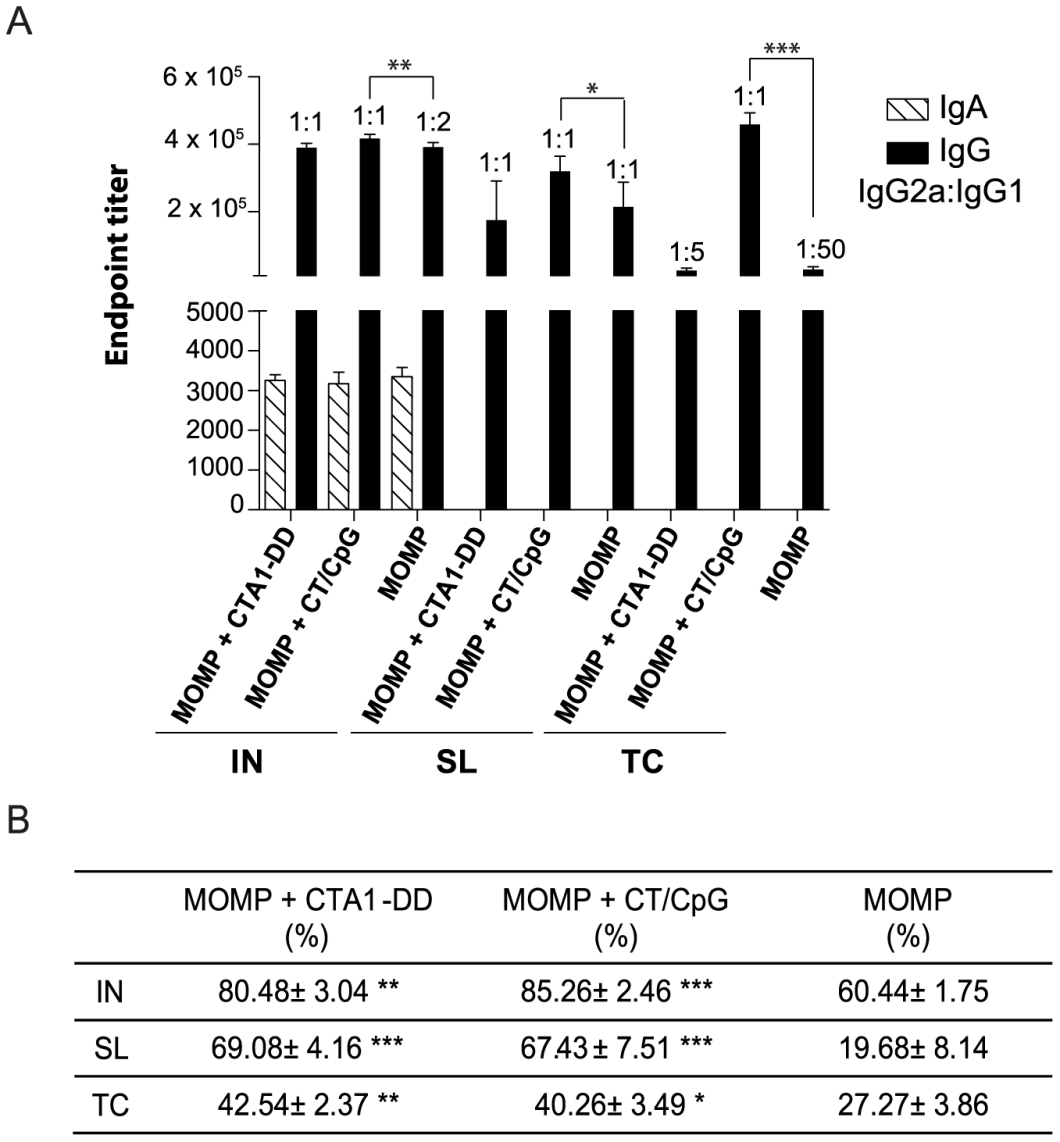
Antigen-specific systemic antibodies in serum. (A) MOMP-specific serum IgG, IgA IgG1 and IgG2a following vaccination was quantified by direct ELISA. Endpoint tires were calculated for all samples using background absorbance of PBST plus two SD. The ratio of IgG2a:IgG1, used to determine Th1:Th2 polarization, is indicated above the titer bars in each group. (B) Percentage of infection neutralized *in vitro* was determined by incubation of *Chlamydia* with a 1/10 dilution of whole serum. Results are presented as the mean ± SD. Significant differences were determined using a one-way ANOVA with Tukey’s post-test. Significance was set at *P*<0.05 for all tests. *P*>0.05 (not shown), 0.01–0.05 (*), 0.001–0.01 (**) and <0.001 (***).

The presence of *Chlamydia*-specific antibodies can also offer protection against the establishment of infection and pathology [29]. Serum IgG levels varied based on both adjuvant and immunization route, but serum IgA production was unique to the IN route and independent of adjuvant (Figure 6). The MOMP and CT/CpG vaccine elicited significantly greater levels of MOMP-specific IgG in serum than MOMP and CTA1-DD by any routes (*P*<0.05–0.001). Consistent with results seen previously [18], the addition of the CTA1-DD adjuvant to a MOMP-based vaccine did not boost antigen-specific antibody titers when compared to the antigen alone controls. Overall, the IN route appeared superior to SL and TC routes for inducing a systemic antibody response. Interestingly, there was no significant difference in IgG titers between immunization with MOMP and CT/CpG via the IN or TC route.

The combination of MOMP and adjuvants CT and CpG induced the desired balanced Th1/Th2 response via all routes, as shown by the IgG2a:IgG1 ratios of 1:1 (Figure 6). Similarly, immunization with MOMP and CTA1-DD also elicited a balanced Th1/Th2 response via the IN and SL route (1:1), but a more Th2-dominant response by the TC (1:5) route. Immunization with the antigen alone, in the absence of a polarizing adjuvant, by the IN (1:2) or TC routes (1:50) elicited, Th2-dominant responses, whereas the SL route elicited a more balanced response (1:1).

Immunization with MOMP together with CTA1-DD or CT/CpG increased (between 10–50%) the *Chlamydia*-neutralizing capabilities of the serum when compared to the antigen only controls (Figure 6). There was however no significant differences in the neutralizing capacities of the serum, between animals immunized with either vaccine via any route. Despite the similarities in MOMP-specific antibody titers between the CTA1-DD-based vaccine and the antigen alone control, there was a significant increase in the neutralizing capabilities of the serum in animals immunized with the MOMP and CTA1-DD (*P*<0.01–0.001). Moreover, there was a significant improvement in the neutralizing potential of serum from animals immunized with MOMP and CT/CpG via the SL compared to TC route (*P*<0.01), despite the latter group inducing significantly higher MOMP-specific IgG titers (*P*<0.01). The IN route was again the most effective when compared to SL and TC routes, for the production of neutralizing antibodies.

### Antigen-specific mucosal antibodies

**Figure 7:**
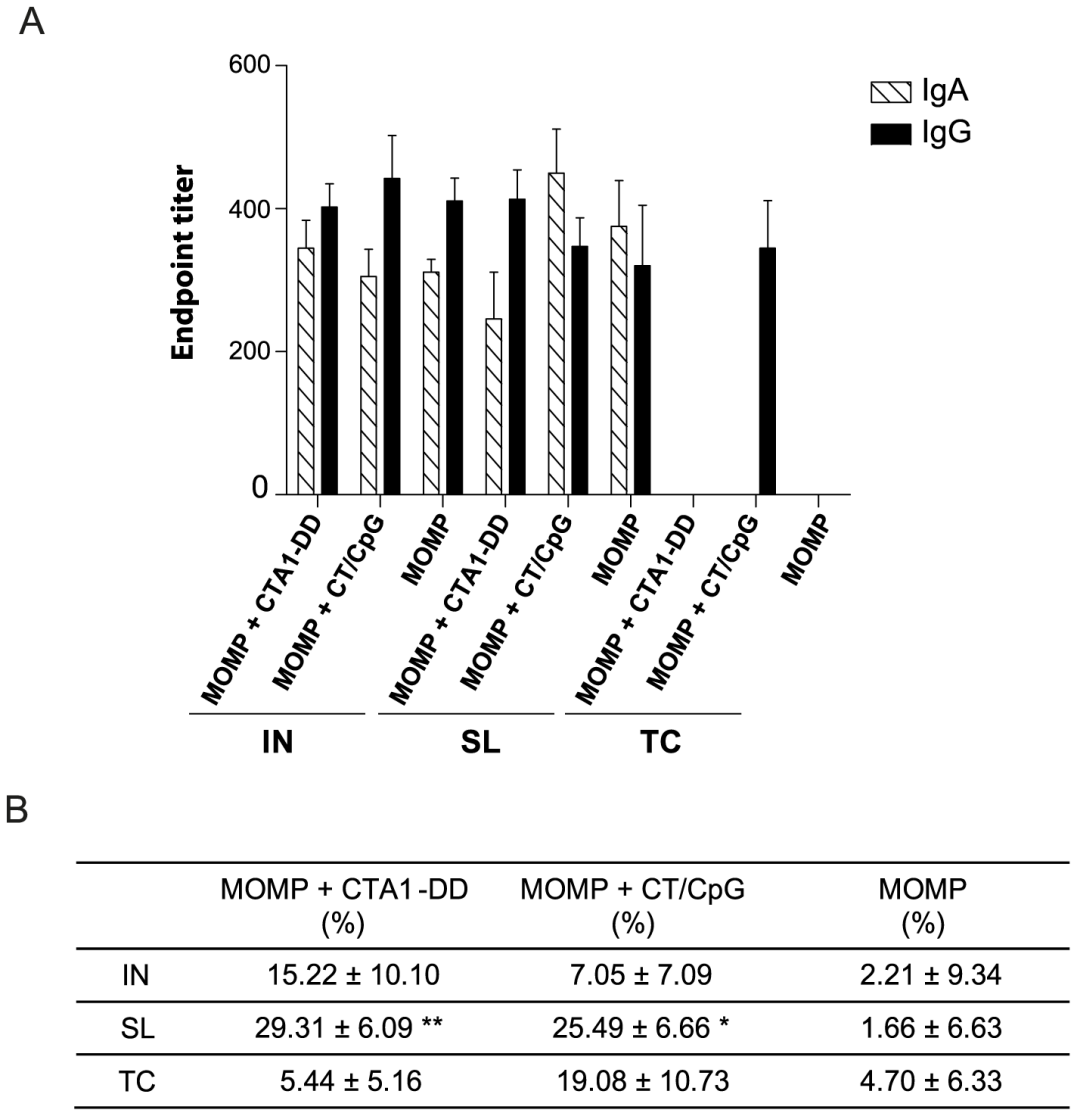
Antigen-specific mucosal antibodies in BAL. (A) The induction of MOMP-specific IgG and IgA in the BAL following vaccination was quantified by direct ELISA. (B) Percentage of infection neutralized *in vitro* was determined by incubation of *Chlamydia* with a 1/10 dilution of BAL. Results are presented as the mean ± SD. Significant differences were determined using a one-way ANOVA with Tukey’s post-test. Significance was set at *P*<0.05 for all tests. *P*>0.05 (not shown), 0.01–0.05 (*), 0.001–0.01 (**) and <0.001 (***).

In the respiratory tract secretions there were no significant differences in antibody titers (IgG or IgA) between animals immunized with MOMP and either CT/CpG or CTA1-DD, with the exception of animals immunized via the TC route (Figure 7). No IgA was detected following TC immunization; however, the MOMP and CT/CpG vaccine did elicit a BAL IgG response by the same route. IgA and IgG production was detected in the mucosal secretions of animals immunized via the IN and SL route. The neutralizing capacity of BAL was enhanced by both adjuvants compared to immunization with MOMP alone, however this was only found to be significant in the BAL of animals immunized via the SL route (*P*<0.05–0.01).

## Discussion

In this study, we assessed the level of protective immunity generated against a chlamydial respiratory tract infection following needle-free immunization with MOMP combined with two different adjuvants. Immunization of animals with MOMP and CT/CpG via the TC and SL routes significantly reduced infection-associated weight loss and the amount of recoverable chlamydial DNA from the lungs at day 10 p.i. This protection was similar to the immunity acquired from a natural infection, but also included near complete protection from pathology (fibrosis and lung consolidation). Interestingly, MOMP and CT/CpG prevented both progressive weight loss and chlamydial burden regardless of administration route. Conversely, MOMP together with CTA1-DD was only protective against the infection following IN administration. Induction of a strong serum IgG and splenic cell-mediated response, dominated by pro-inflammatory cytokines IFNγ, TNFα, IL-17, was associated with the protection against infection. A local pro-inflammatory cell-mediated response in the lymph nodes draining the lungs however, determined whether fibrotic scarring was prevented. Interestingly, the protection against infection elicited by both IN delivered vaccines (reduced weight loss and bacterial load) did not however, provide protection from pulmonary fibrosis. Moreover, TC immunization with MOMP and CTA1-DD prevented pathology despite having no significant influence on chlamydial burden and a minimal effect on weight loss. Therefore, this study not only identified vaccinations that protect against both infection and pathology, but also infection or pathology individually. This illustrates the challenges involved when developing a vaccine against *Chlamydia*: should the primary aim be to prevent infection (sterilizing immunity) or prevent disease-associated pathology, as reducing the chlamydial burden in the lungs does not necessarily protect against pathology and vice versa, at least in this mouse model.

The route of immunization influenced the effectiveness of each adjuvant; particularly CTA1-DD. IN immunization with either vaccine elicited similar MOMP-specific immune responses. However, immunization with MOMP and CT/CpG consistently elicited stronger antigen-specific cell-mediated and humoral responses than the CTA1-DD-containing vaccine, when both vaccines were administered by the SL or TC routes. This difference is likely linked closely with each adjuvant’s mechanism of action, as well as the antigen-presenting cells (APCs) targeted and their density/distribution at the site of immunization. CpG activate toll-like receptor (TLR) 9 that is expressed by keratinocytes, Langerhan’s cells, plasmacytoid dendritic cells (DCs), myeloid DCs, macrophages/monocytes, mast cells and B cells in mice, which elicit a Th1-polarized response upon activation through TLR9 [30]. CT binds all nucleated mammalian cells via the GM_1_-ganglioside receptor to enhance Th2 as well as Th1 responses [31]. Together, both CpG and CT elicit a balance between Th1 cell-mediated and Th2-driven humoral immunity, crucial for a chlamydial vaccine. The success of CT/CpG by IN, SL or TC routes of administration could be attributed to the adjuvants’ capacity to target and stimulate a number of innate cell populations localized at the site of immunization, thus enhancing antigen uptake/presentation and the development of adaptive immunity.

CTA1-DD is a non-toxic derivative of CT that retains the immunomodulatory properties of the holotoxin i.e. Th1/Th2 polarizing response, but differs greatly in target cell binding. CTA1-DD is thought to predominantly, but not exclusively, target B cells as APCs [11]. Recently, CTA1-DD has been shown to bind follicular DCs in B cell follicles in a CR2-dependent manner, to enhance germinal centre reactions and T cell-dependent responses by activation of the complement pathway [32]. The immunogenicity of CTA1-DD, similar to CT, is highly dependent on binding and internalization of the enzymatically active ADP-ribosyltranferase subunit for optimal adjuvanticity [33]. This could explain why CTA1-DD was immunogenic via the IN route, which targets the B cell and follicular DC rich nasopharynx-associated lymphoid tissue, but was less effective when administered via the TC and SL, routes targeting tissues where these target cells are not normally found in great numbers in a resting state [34], [35].

In this study, the vaccinations that induced a significant amount of protection from a respiratory tract infection, determined by weight loss and chlamydial burden, generated the highest titers of MOMP-specific serum antibodies. Serum antibodies have been shown to neutralize *Chlamydia in vitro* and *in vivo* [36]. Additionally, B cell-deficient mice challenged IN with *C. muridarum* have also been shown to be more susceptible to re-infection, have higher mortality rates and develop more severe cachexia compared to WT mice [28], [29]. This suggests that antibodies play an important role in preventing and controlling infection in the chlamydial respiratory tract model.

We also found that MOMP-specific IgG, and not IgA, was a better associate of protection against a respiratory infection *in vivo*. IgA has previously been shown to have no effect on the resolution of a primary respiratory tract infection or the incidence of mortality following passive immunization [37]. The IgA generated in the serum following vaccination in our study, significantly improved its neutralization of *Chlamydia in vitro*. However, this IgA may not have possessed the necessary secretory component required by the polyclonal immunoglobulin receptor (pIgR) to transcytose IgA into the lumen to neutralize the infection *in vivo*, by preventing microbial attachment. In addition, the expression of pIgR has been shown to be absent in the murine lungs [38], suggesting that even secretory IgA may be unable to enter the respiratory tract effectively. The lack of protection offered by IgA in this study can be supported by the detection of low levels of IgA in the BAL, which did not enhance neutralization *in vitro* or associate with protection against infection *in vivo*. Therefore, IgA was ineffective at mediating protection against infection *in vivo*, possibly due to the induction of insufficient levels of IgA in the mucosal secretions needed to inhibit chlamydial attachment. Alternatively, IgG is able to passively transfuse from the serum into the highly vascularized lung mucosal tissues. The inability of IgG alone to neutralize *Chlamydia* infectivity *in vitro* as effectively as IgA however, suggests that the protection mediated by IgG *in vivo* may occur through Fc-dependent mechanisms as opposed to direct neutralization [39].

Despite the positive influence that high serum antigen-specific IgG titers had on protection against infection, the quantity, quality or class of antibodies in the serum or mucosal secretions could be associated with protection from pathology. IgA-deficient mice display exaggerated lung histopathology following an IN infection, even in the presence of a compensatory over-production of other serum antibodies [37]. This suggests that IgG is ineffective at preventing pathology, while IgA plays a role in regulating pulmonary inflammation and mucosal homeostasis following a primary chlamydial respiratory infection. The low levels of MOMP-specific IgA detected in the BAL following vaccination, which failed to neutralize the infection *in vitro* or provide protection against infection *in vivo*, may have also been unable to regulate inflammation and hence prevent pathology. Moreover, suboptimal concentrations of IgG have even been associated with enhancement of pathology [40]. MOMP-specific antibodies induced following vaccination were therefore unable to prevent the development of pathology following a respiratory tract challenge, despite their positive influence on infection.

Surprisingly, IN immunization with MOMP alone generated strong antigen-specific humoral and cell-mediated (preferentially IL-17) responses that provided partial protection against the infection. Genetically linking the MOMP with MBP improves the solubility and correct conformational folding of the recombinant protein [41], [42], vital for production of neutralizing antibodies [43]. However, MBP can also acts as an adjuvant [44]–[48]. Proteins fused with MBP elicit significantly more serum antibodies than untagged proteins [45]–[47], as MBP is a TLR4 agonist that stimulates cytokine production and expression of co-stimulatory molecules on DCs [44]. This provides an explanation for why IN immunization with recombinant MOMP, in the absence of an adjuvant, elicits a strong antigen-specific response as opposed to mucosal tolerance [49], [50]. Furthermore, Zygmunt *et al*., (2009) described a predisposition for immunization via the IN route to promote Th17 immune responses due to an adjuvant-independent over production of IL-6 by DC localized in the nasopharynx-associated lymphoid tissue [51], which would also explain the preferential induction of IL-17 when antigen is delivered intranasally.

The vaccinations that elicited a significant level of protection against weight loss and infectious burden following an respiratory infection, induced secretion of the pro-inflammatory cytokine TNFα, IL-17 and IFNγ by splenocytes following *in vitro* re-stimulation with MOMP. These same immunizations also elicited low-level secretion of the anti-inflammatory cytokines IL-4 and IL-10 by splenocytes. As the induction of pro-inflammatory cytokines (IFNγ, TNFα and IL-17) by T cells is known to enhance the resolution of infection [28], [52]-[55] and anti-inflammatory cytokines (IL-4 and IL-10) inhibit the clearance of a chlamydial respiratory tract infection [28], [52], these vaccinations primed the ideal response necessary to eradicate an active infection.

Pro-inflammatory cytokines, IFNγ, TNFα and IL-17 have been shown to act individually and synergistically to improve inhibition of chlamydial growth. IFNγ-mediated inhibition of *Chlamydia* growth *in vitro* is enhanced 2-fold in the presence of TNFα [56], [57]. The synergism between IFNγ and TNFα is likely to be heightened further *in vivo*, as TNFα-mediated inhibition of chlamydial growth is indirect and requires the recruitment of additional cell populations [58]. TNFα and IFNγ can also synergize with IL-17 to up-regulate intercellular adhesion molecule 1 [59], [60], which is associated with immune cell recruitment, activation of the Th1 response and the normal resolution of a chlamydial genital tract infection [61], [62]. In addition, the induction of CD4^+^ T cells secreting a combination of cytokines (IFNγ^+^TNFα^+^ and IL-17^+^IFNγ^+^ double positives, specifically) following vaccination has been shown by others as an excellent correlate of protection against a chlamydial genital tract infection [63], [64]. Therefore, the production of IFNγ, TNFα and IL-17 following immunization coincided with strong protection against infection, potentially due to a synergistic interaction between multiple pro-inflammatory cytokines. To the best of our knowledge, this is the first evidence of this combination cytokine response, first mentioned by Igiesteme *et al*., (1993) and Yu *et al*., (2010), conferring protection against *Chlamydia* in the respiratory tract infection model. Although we cannot definitively say whether CD4^+^ T cells were co-producing these cytokines upon re-stimulation with MOMP, the quality of T cells and the development of a multi-functional phenotype can be influenced by the adjuvant [64], duration of antigen exposure [65], the type of APCs targeted [66] and the innate cytokine milieu at the site of immunization [67]

Unexpectedly, we were unable to detect an IFNγ response in the spleen or MdLN following SL immunization with MOMP plus CT/CpG, even though this group of animals was significantly protected against infection. It is possible that IFNγ-secreting MOMP-specific cells were present locally, instead adopting a more effector phenotype and migrating to other non-lymphoid tissues [68]. Alternatively, the lymphocytes may have produced IFNγ following antigenic stimulation at a point in time not chosen for cytokine analysis [69].

Our data indicated that protection against infection was associated with a strong pro-inflammatory cell-mediated response in the spleen. TNFα and IFNγ are known to be potent inhibitors of fibrosis [70], although the production of these cytokines by splenocytes in this study could not be linked with protection against pathology. However, when a significant level of local antigen-specific proliferation and cytokine production was detected in the MdLN draining the respiratory tract, protection against infection increased significantly and extended to include the prevention of pathology. The enhancement of protection against infection and pathology seen in these groups may reflect the ability of an antigen-specific cell-mediated response residing in the lymph nodes draining the site of infection to respond more rapidly than a splenic response [71], [72]. Support for this hypothesis is provided by our findings that groups mounting a local response in the lymph nodes draining the site of infection following vaccination (TC or SL immunization with MOMP and CT/CpG), were more resistant to weight loss earlier during the course of infection, when compared to animals IN immunized with MOMP and CT/CpG, that only mounted a systemic response. Therefore, protection against pathology following a respiratory challenge with *Chlamydia* may rely on a vaccine priming and positioning a cell-mediated response in the regional lymph nodes, that can respond early during an infection and potentially limit the involvement of the innate response in eradicating the infection.

Interestingly, there appeared to be a significant disconnection between infection and pathology in some vaccine groups, where a reduction in chlamydial burden did not necessarily coincide with protection against inflammatory disease and vice versa. Both IN delivered vaccines elicited protection from infection, yet seemed to exacerbate pathology compared to that of a normal course of infection. IN immunization elicited the strongest pro-inflammatory cytokine response that may have caused an imbalance between the control of infection and prevention of disease, resulting in excessive immunopathology [73]. Alternatively, it is conceivable that trace amounts of the vaccine may have drained into the lungs [74] and caused local inflammation and fibrosis, interpreted as infection-induced pathology.

This does not appear to be the case for the TC delivered CTA1-DD-adjuvanted vaccine group, which was solidly protected from pathology despite having no significant reduction in the infectious burden. Although the underlying mechanisms in this case are unknown, it has been suggested that inducible tolerogenic pathways protective against disease could exist for many types of infection, including *Chlamydia* [75]. Identifying and understanding these pathways may form the basis for new treatment strategies that could include vaccines.

In conclusion, the CTA1-DD adjuvant has been shown to elicit strong protection against a number of respiratory tract pathogens [13], [15], which now includes *Chlamydia*. CTA1-DD also represents the next generation of non-toxic mucosal adjuvants found to be safe in primates [12] and its adjuvanticity and safety in humans is currently under investigation. The CT/CpG adjuvant combination could potentially be used for TC immunization in humans. The TC route would minimize the toxicity associated with IN and SL delivery of CT in humans. Alternatively, replacing CT with CTA1-DD may allow the safe use of a CTA1-DD/CpG combination, delivered by either the SL or IN routes, in humans. Studies are currently in progress to evaluate the effectiveness of this adjuvant combination in our animal model. The induction of high serum MOMP-specific IgG, but not IgA, was associated with protection against infection and this was largely mirrored in the mucosal secretions. Systemic pro-inflammatory cell-mediated responses were also associated with the enhanced resolution of infection, possibly due to the synergistic effect of IFNγ, TNFα and IL-17. Detection of antigen-specific cell-mediated responses in the lymph nodes draining the lungs were associated with protection against pathology, which may reflect the time required to initiate and recruit an immune response to the site of the infection. Interestingly, protection against pathology developed in cases when the chlamydial DNA recovered from the lung tissue was unaltered. This suggested that there may be other immunopathological mechanisms modulating the development of disease following infection, which are independent of infection control and could be potential targets for future vaccines.
